# Pullulan Films Containing Rockrose Essential Oil for Potential Food Packaging Applications

**DOI:** 10.3390/antibiotics9100681

**Published:** 2020-10-08

**Authors:** Ângelo Luís, Ana Ramos, Fernanda Domingues

**Affiliations:** 1 Centro de Investigação em Ciências da Saúde (CICS-UBI), Universidade da Beira Interior, Avenida Infante D. Henrique, 6200-506 Covilhã, Portugal; fdomingues@ubi.pt; 2 Laboratório de Fármaco-Toxicologia, UBIMedical, Universidade da Beira Interior, Estrada Municipal 506, 6200-284 Covilhã, Portugal; 3 Departamento de Química, Faculdade de Ciências, Universidade da Beira Interior, Rua Marquês d’Ávila e Bolama, 6201-001 Covilhã, Portugal; ammr@ubi.pt; 4 Materiais Fibrosos e Tecnologias Ambientais (FibEnTech), Universidade da Beira Interior, Rua Marquês d’Ávila e Bolama, 6201-001 Covilhã, Portugal

**Keywords:** pullulan, rockrose, essential oil, *Cistus ladanifer*, α-pinene, bioactive films, food packaging, *quorum sensing*, biofilms

## Abstract

Active packaging is designed to control the development of decay- and disease-causing microorganisms and is emerging as a promising technology for extending shelf-life, maintaining food safety, reducing waste, and minimizing the risks for foodborne diseases. The goal of this work was to develop and characterize bioactive pullulan-based films, containing rockrose (*Cistus ladanifer*) essential oil. Among other abundant compounds (camphene, bornyl acetate and *trans*-pinocarveol), α-pinene was identified as the major compound of rockrose essential oil (39.25%). The essential oil presented stronger antibacterial activity against Gram-positive than against Gram-negative bacteria. The antioxidant results indicate the potential of the developed films to be used to package foods susceptible to oxidation and rancification, thus improving their shelf-life. Also, this study reflects the potential of rockrose essential oil, free or incorporated in pullulan, as a promising *quorum sensing* inhibitor, since it was able to interrupt intercellular communication, inhibiting violacein production. Electronic microscopy images showed the antibiofilm activity of the films with rockrose essential oil that were able to influence bacterial adhesion, which may be explained by the differences in the surface free energy of the films, as also determined.

## 1. Introduction

There is growing worldwide interest in replacing petrochemical-based, synthetic plastic packaging with biodegradable, nontoxic and edible materials. This development of new packaging materials can benefit several industrial activities, particularly food production, distribution, commercialization and preservation [1,2,3]. As potential replacements for conventional plastics, biopolymers such as polysaccharides, proteins and lipids can be applied for the sustainable development of packaging materials [2,3,4].

Food safety is an important global concern with health and trade implications. According to the Centers for Disease Control and Prevention, foodborne diseases account for approximately 48 million illnesses, 128,000 hospitalizations, and 3000 deaths each year in USA [5]. Undesirable microbial growth and oxidative reactions (biochemical and enzymatic) are responsible for most spoilage in meat, poultry products and other refrigerated foods [5].

Active packaging, which is designed to control the development of decay- and disease-causing microorganisms, emerges as a promising technology for extending shelf-life, maintaining food safety, reducing waste and minimizing the risks for foodborne diseases [6,7,8]. Within this new food packaging technology, the active agent is incorporated within a suitable polymeric matrix from which it is released following diffusion mechanisms and accumulated into the food packaging system following thermodynamic principles [9,10].

Pullulan is a nonionic, linear, water-soluble and neutral polysaccharide composed of α-(1, 6) repeated maltotriose units via α-(1, 4) glycosidic bonds [11]. Pullulan is widely used as a film-forming agent in the preparation of a variety of thin films due to its properties, namely, impermeability to oxygen and nonhygroscopic and nonreducing capacities. It is easily soluble in water to make a clear and viscous solution and has excellent adhesion and film-forming abilities. The principal advantages of pullulan are that it is a nonionic polysaccharide, which is blood compatible, biodegradable, nontoxic, nonimmunogenic, nonmutagenic and noncarcinogenic [12].

Essential oils are secondary metabolites of aromatic plants, which can be used as natural antioxidant and antimicrobial substances. The antimicrobial effects of these volatile mixtures of compounds results from their ability to increase permeability, leading to the loss of cellular constituents of microorganisms [13]. *Cistus ladanifer* (“esteva”), also known as rockrose, is a perennial Mediterranean shrub widely distributed in the Iberian Peninsula [14,15], and the main species producing labdane, a resin employed as a natural fixative and as a fragrance for composing amber and leathery notes [16]. Other odoriferous materials may be obtained from fresh leaves and branches, namely, rockrose essential oil (EO) by steam distillation, cistus concrete by nonpolar solvent extraction, and absolute by taking up concrete [16]. Most studies on the chemical profile characterization of *C. ladanifer* have mainly focused on its EO (cistus oil), restricting analyses to mono- and sesquiterpenes, its main constituents [17]. Rockrose EO is known for its bioactivities, such as acting as preservative against fungal and aflatoxin B_1_ contamination [18].

The goal of this work was to develop and characterize bioactive pullulan-based films, containing rockrose EO. The chemical composition of the EO was evaluated by gas chromatography-mass spectrometry (GC-MS) and gas chromatography-flame ionization detector (GC-FID) analyses. The antioxidant and antibacterial activities of the developed films were assessed, together with their antibiofilm properties.

## 2. Materials and Methods

### 2.1. Essential Oil

Rockrose (*Cistus ladanifer*, *Cistaceae*) EO (pure and biologic) was obtained from Herdade de Vale Côvo (Parque Natural do Vale do Guadiana, Serra de Mértola, Alentejo, Portugal). The EO was isolated from the aerial parts of the shrub, particularly the leaves, which grows spontaneously in the field (biologic agriculture, PT-BIO-02, ECOCERT, Portugal), harvested by hand and immediately subjected to steam-distillation in a stainless-steel alembic. The rockrose EO was stored at −20 °C in the dark until analysis and further use. The purity of the EO was tested and its quality assured for use by humans.

### 2.2. Essential Oil Chemical Analysis

The compounds of rockrose EO were identified by GC-MS, being processed in “total ion chromatogram” (TIC) mode, and quantified by GC-FID, using the relative area percentage.

An Agilent 7820A GC-FID (Santa Clara, CA, USA) equipped with an Agilent 5977B MS (Santa Clara, CA, USA) detector, along with a HP-INNOWax column (Agilent, Santa Clara, CA, USA) (60 m × 0.25 mm × 0.5 µm), were used. The oven temperature was programmed for 6 min at 50 °C, 2 °C/min to 190 °C, 4 °C/min to 220 °C, 10 min at 220 °C, 4 °C/min to 250 °C and finally 10 min at 250 °C. The carrier gas was helium at a head pressure of 33 Psi (FID) and 25.5 Psi (MSD), with an injection volume of 0.1 µL for FID and 0.1 µL for MSD, using split mode.

### 2.3. Antioxidant Activity Evaluation

#### 2.3.1. DPPH Free Radical Scavenging Assay

Briefly, 0.1 mL of several concentrations of rockrose EO were mixed with 3.9 mL of three DPPH (2,2-diphenyl−1-picrylhydrazyl) (Sigma-Aldrich, St. Louis, MO, USA) methanolic solutions (0.20, 0.12 and 0.08 mM). The negative control was composed of 0.1 mL methanol and 3.9 mL of each DPPH solution. Gallic acid (Sigma-Aldrich, St. Louis, MO, USA) was used as positive control. After incubation for 90 min at room temperature in the dark, the absorbances were measured at 517 nm using a spectrophotometer (Helios-Omega, Thermo Scientific, Waltham, MA, USA). The radical scavenging activity was calculated using the Equation (1) [19]:

(1) $$\%Inhibition=\frac{A_{control}\;-\;A_{sample}}{A_{control}}\times 100$$

where *A_control_* is the absorbance of the negative control and *A_sample_* is the absorbance in the presence of the EO.

The IC_50_ was determined using a calibration curve by plotting the rockrose EO concentrations versus the corresponding %Inhibition. The antioxidant activity was expressed as the antioxidant activity index (AAI), calculated as follows (Equation (2)) [19]:

(2) $$AAI=\frac{final\;concentration\;of\;DPPH\;in\;the\;negative\;control}{IC_{50}}.$$

The AAI allowed the classification of the antioxidant activity of the EO as: poor (AAI ≤ 0.5), moderate (0.5 < AAI ≤ 1.0), strong (1.0 < AAI < 2.0) or very strong (AAI ≥ 2.0) [20]. The assays were carried out in triplicate and the DPPH solutions were prepared daily.

#### 2.3.2. β-Carotene Bleaching Test

Initially, 500 μL of a β-carotene (Sigma-Aldrich, USA) solution (20 mg/mL in chloroform) was added to 40 μL of linoleic acid (TCI Europe N.V., Belgium), 400 μL of Tween 40 (Riedel-de Häen, Germany) and 1 mL of chloroform (Scharlab, Spain). The chloroform was then evaporated under vacuum, and 100 mL of distilled water saturated with oxygen were added to the mixture to form an emulsion. Then, 5 mL of this emulsion was pipetted into test tubes containing 300 μL of methanolic dilutions of rockrose EO. The negative control consisted in 5 mL of the emulsion and 300 μL of methanol, and the reference antioxidant butylated hydroxytoluene (BHT) (Sigma-Aldrich, St. Louis, MO, USA) was used as positive control. Finally, the tubes were shaken and placed at 50 °C in a water bath for 1 h. The absorbance of the samples was measured at 470 nm, using a spectrophotometer (Helios-Omega, Thermo Scientific, Waltham, MA, USA), against a blank containing an emulsion without β-carotene. The measurements were carried out in triplicate at the initial time (t = 0 h) and at the final time of incubation (t = 1 h). The antioxidant activity of the EO was determined as the percentage of inhibition of β-carotene oxidation (%Inhibition) by the Equation (3) [19]:

(3) $$\%Inhibition=\frac{A_{sample}^{t=1h}-A_{control}^{t=1h}}{A_{control}^{t=0h}\;-\;A_{control}^{t=1h}}\times 100$$

where *A^t = 1 h^* is the absorbance of the sample or negative control at the final time of incubation, and *A^t = 0 h^* is the absorbance of the negative control at the initial time of incubation.

### 2.4. Antibacterial and Anti-quorum sensing Properties Assessment: Solid Diffusion Assay

The antibacterial activity of rockrose EO was evaluated against seven bacterial species: four Gram-positive (*Staphylococcus aureus* ATCC 25923, *Listeria monocytogenes* LMG 16779, *Enterococcus faecalis* ATCC 29212 and *Bacillus cereus* ATCC 11778), and three Gram-negative (*Escherichia coli* ATCC 25922, *Salmonella* Typhimurium ATCC 13311 and *Pseudomonas aeruginosa* ATCC 27853). Stock cultures of the bacterial strains were prepared and kept with 20% (*v/v*) glycerol at −80 °C. All the strains were subcultured in brain-heart infusion agar (BHI) (Liofilchem, Italy) for 24 h before the antibacterial assays.

For the solid diffusion assay, inoculums were prepared by suspending bacteria in a sterile saline solution (NaCl, 0.85% *w/v*) to a cell suspension of 0.5 McFarland (1–2 × 10^8^ colony-forming units/mL (CFU/mL)). Sterile blank filter discs with a diameter of 6 mm (Filtres Fioroni, Ingré, France) were saturated with 15 μL of rockrose EO. Then, the Müeller-Hinton agar (MHA) (Liofilchem, Italy) plates were inoculated and allowed to dry, and the previously prepared discs were placed over the inoculated culture medium. Finally, the plates were incubated at 37 °C for 24 h. After the incubation, all the plates were visually checked for inhibition zones, i.e., their diameters were measured using a digital pachymeter. This assay was performed three times, using dimethyl sulfoxide (DMSO) (15 μL/disc) (Sigma-Aldrich, St. Louis, MO, USA) as negative control, and tetracycline (30 μg/disc) (Sigma-Aldrich, St. Louis, MO, USA) as positive control [19].

The biomonitor strain *Chromobacterium violaceum* ATCC 12472 was used to assess the anti-*quorum sensing* activity of rockrose EO. The bacterial suspension of *C. violaceum* ATCC 12472 was obtained by overnight aerobic growth (30 °C, 250 rpm) in Luria–Bertani (LB) (Liofilchem, Italy) broth. The turbidity of *C. violaceum* ATCC 12472 suspension was adjusted to an OD_620 nm_ of 1 and then seeded in LB agar (Liofilchem, Italy) plates. Sterile discs saturated with 15 μL of the EO were placed over the plates and incubated (30 °C, 24 h). After the incubation period, the anti-*quorum sensing* activity, evaluated by the inhibition of the violacein pigment production around the disc (a ring of colorless but viable cells), was measured with a digital pachymeter. These experiments were performed in three independent assays using DMSO (15 μL/disc) and resveratrol (5 μg/disc) (TCI Europe N.V., Belgium) as negative and positive controls, respectively [21].

### 2.5. Determination of MIC Values: Resazurin Microtiter Method

The values of minimum inhibitory concentrations (MIC) of rockrose EO were determined using the resazurin microtiter assay. Serial two-fold dilutions of EO (from 32 to 0.25 μL/mL) were prepared in a 96-well plate (50 μL/well) using Müeller–Hinton broth (MHB) (Liofilchem, Italy) as the culture medium and with a maximum DMSO final concentration of 2% (*v/v*) to increase the solubility of the EO. Then, resazurin (TCI Europe N.V., Belgium) indicator solution (10 μL, 0.1% *w/v* diluted in MHB) was added to each well, followed by the addition of 30 μL of fresh MHB. Finally, a bacterial suspension (10 μL, 0.5 McFarland units, 1–2 × 10^7^ CFU/mL per well) was added to the wells (final volume: 100 μL/well). Each plate had a set of controls: a column with tetracycline as a positive control, a column with all solutions except the test compounds, and a column with all solutions except the bacterial suspensions, adding the respective volume of MHB instead. The plates were prepared in triplicate and incubated at 37 °C for 24 h. The color change from purple to pink or colorless was then assessed visually and recorded as positive. The lowest concentration at which the color change occurred was taken as the MIC value [21].

### 2.6. Preparation of Bioactive Films

The bioactive films were prepared by the solution casting method. Initially, the pullulan (CAS Number: 9057-02-7) (TCI Europe N.V., Belgium) solution (3%, *w/v*) was prepared by dissolving pullulan powder in deionized water at room temperature for 5 min under magnetic stirring. After that, 15% (*w/w* pullulan) glycerol (anhydrous extra pure) (Merck, Darmstadt, Germany) was added as a plasticizer and stirred at 50 °C for 30 min. The rockrose EO (15%, *w/w* pullulan) was then added to the film-forming solution, which was stirred again at 50 °C for 10 min. Finally, this mixture was homogenized for 4 min at 7600 rpm and for another 4 min at 12,000 rpm using a rotor/stator homogenizer (IKA T25 Digital Ultra-Turrax, Staufen, Germany). To obtain the bioactive films, ≈16 mL of the film-forming solution were casted over polystyrene Petri dishes and dried at 60 °C for approximately 2 h in a ventilated oven. Control films without the addition of rockrose EO were also prepared. The dried films were peeled off and stored in a laboratory with both controlled temperature (23 ± 2 °C) and relative humidity (RH) (50 ± 5%) [22].

### 2.7. Characterization of Films

#### 2.7.1. Infrared Spectra

FTIR (Fourier-Transform Infrared Spectroscopy) spectra of bioactive films were obtained between 4000 and 600 cm^−1^ using a Nicolet iS10 smart iTRBasic (Thermo Fisher Scientific, Waltham, MA, USA) model, with 64 scans and a 4 cm^−1^ resolution [23].

#### 2.7.2. Thermal Analysis

DSC (Differential Scanning Calorimetry) analysis of the films was performed on a calorimeter (Netzsch DSC 204, Selb, Germany) operating in the following conditions: heating rate of 5 °C/min, inert atmosphere, and temperature range from 25 to 500 °C. Before the analysis, the bioactive films were placed at 105 °C for 24 h to completely evaporate the water, and the respective baselines were obtained [24].

#### 2.7.3. Grammage, Thickness, Mechanical and Optical Properties

The grammage of the films was calculated based on the ratio between their mass and area (g/m^2^), following the ISO 536:1995. The thickness (µm) was measured according to ISO 534:2011 using a micrometer (Adamel Lhomargy Model MI 20, Veenendaal, Netherlands), considering several random measurements [23].

The mechanical properties of the films (elongation at break (%), tensile index (N.m/g) and elastic modulus (MPa)) were obtained using a tensile tester (Thwing-Albert Instrument Co., West Berlin, NJ, USA), following the ISO 1924/1, with the initial grip set at 50 mm and the crosshead set at 10 mm/min [23]. 

The color and transparency of the films were assessed using a Technidyne Color Touch 2 spectrophotometer (New Albany, IN, USA). These measurements were performed on several random positions of the films using the illuminant D65 and the observer 10°. Color coordinates L* (lightness), a* (redness; ±red-green) and b* (yellowness; ±yellow-blue) were obtained [23].

#### 2.7.4. Contact Angles and Surface Free Energy

The contact angles of the films were determined by the sessile drop contact angle method using a model OCAH 200 (DataPhysics Instruments, Filderstadt, Germany) that allowed image acquisition and data analysis. The surface free energy (total, dispersive and polar components) of the films was determined by measuring the contact angles with three pure liquids (deionized water, ethylene glycol and diiodomethane) [25]. The surface tension components of the reference liquids were provided by the equipment’s software [26]. Contact angle data were obtained from at least three determinations for each liquid and for each sample, and the surface free energies of the samples were obtained employing the Owens-Wendt approach [27].

#### 2.7.5. Barrier Properties 

##### Water Vapor Permeability

Water vapor permeability (WVP) (g/Pa.day.m) and water vapor transmission rate (WVTR) (g/m^2^.day) were determined according to the standard protocol ASTM E96-00. The films were fixed on the top of equilibrated cups containing a desiccant (15 g of anhydrous CaCl_2_, dried at 105 °C before being used). The test cups were then placed in a cabinet at 23 ± 2 °C and 50 ± 5% RH. The weight changes were monitored every 2 h over a period of 48 h. The gradient was calculated from the slope of a linear regression of the weight increase versus time. WVTR and WVP were calculated according to the Equations (4) and (5) [23]:

(4) $$WVTR=\frac{\frac{\Delta m}{\Delta t}}{A}$$

where ∆*m* is the weight changes of test cups (g), *A* is the test area (m^2^), and *t* is the test time (day).

(5) $$WVP=\frac{WVTR}{\Delta p}=\frac{WVTR}{p\times \left(RH_{1}\;-\;RH_{2}\right)}\times e,\;$$

where *p* is the vapor pressure of water at 23 °C (Pa), RH_1_ is the RH of the cabinet (50%), RH_2_ is the RH inside the cups (0%), and *e* is the thickness (m) of the films.

##### Oxygen Permeability

Oxygen transmission rate (OTR) (cm^3^/m^2^.day) was measured according to ISO 15101-2:2003, using the equipment Labthink PERME^®^ OX2/230 (Jinan, China). The films with an exposed area of approximately 5 cm^2^ were clamped into the diffusion cell. Pure oxygen was introduced into the outside chamber of the diffusion cell, and the permeation rate through the sample was measured until a steady state was reached. Oxygen permeability (OP) (cm^3^.µm/m^2^.day.kPa) was achieved by normalizing OTR with respect to the oxygen pressure (1 atm) and the thickness of the samples, using the Equation (6) [28,29]:

(6) $$OP=\frac{OTR}{\Delta pO_{2}}\times e$$

where *e* is the thickness of the films (µm), and ∆*p*O_2_ is the difference of oxygen partial pressure (Pa) between the two sides of the film.

These measurements were performed in duplicate for each sample at 23 ± 0.5 °C and 50 ± 2% RH.

#### 2.7.6. Antioxidant Activity

The antioxidant activity of bioactive films was evaluated by both methods described above for rockrose EO, with slight modifications. For the DPPH free radical scavenging assay, three discs of the films (6 mm of diameter) were added to 2.9 mL of a DPPH methanolic solution (0.1 mM). Then, the absorbances were measured at 517 nm every 30 min, for 5 h, against a blank of methanol. The control sample consisted of 100 μL methanol plus 2.9 mL of the DPPH solution. Using the β-carotene bleaching test, the volume of the sample was substituted by three discs of the films (6 mm of diameter); the remaining protocol was the same used for the EO [19].

#### 2.7.7. Antibacterial and Anti-*quorum sensing* Properties

The antibacterial and anti-*quorum sensing* properties of pullulan-based films were evaluated by the solid diffusion assay, using the methods described above for rockrose EO. Briefly, discs of the films (6 mm of diameter) were prepared under aseptic conditions and placed on the surface of seeded agar plates as previously described [19,27].

#### 2.7.8. Antibiofilm Activity

The antibiofilm activity of the films was studied for the four Gram-positive species of bacteria with the biofilms, observed by scanning electron microscopy (SEM). For that, bacterial biofilms were formed directly on the discs of the films (≈1 cm^2^) placed on 12-well plates. *L. monocytogenes* LMG 16779 and *E. faecalis* ATCC 29212 were gown overnight (37 °C, 250 rpm) in Tryptic Soy Broth (TSB) (Merck, Darmstadt, Germany), while *S. aureus* ATCC 25923 and *B. cereus* ATCC 11778 were grown in MHB. The turbidity of the suspensions was adjusted to an OD_610 nm_ of 0.7. Then, 300 μL of these bacterial suspensions was added to 700 μL of an appropriate culture medium. The plates were incubated at 37 °C for 24 h. Then, biofilms were washed twice with sterile saline solution and fixed with 2.5% (*v/v*) glutaraldehyde (Sigma-Aldrich, USA) at 4 °C for 4 h. After that, samples were washed once with phosphate buffer saline (PBS) and dehydration was carried out in ethanol series for 20 min each (30, 50, 70, 80, 90% (*v/v*), and absolute). The samples were then allowed to dry overnight in a desiccator. Subsequently, they were placed on appropriate stubs and spray-coated with gold using a metal evaporator (Quorum Q150R ES, East Sussex, UK). Finally, biofilms were analyzed by VP SEM Hitachi S–3400N using a voltage of 20.0 kV and 120.0 μA emission [19].

### 2.8. Statistical Analysis

The results were generally expressed as mean ± standard deviation (SD). The data were analyzed using the statistical program IBM SPSS Statistics 25. Significant differences among means were analyzed by Student’s T-test (assuming the normal distribution of the continuous variables). A level of *p*-value < 0.05 was considered significant.

## 3. Results and Discussion

### 3.1. Chemical Composition of Rockrose EO

A chemical analysis of rockrose EO allowed us to identify 95.36% of the compounds, as presented in Table 1. Among other abundant compounds (camphene, bornyl acetate and trans-pinocarveol), α-pinene was identified as the major compound of rockrose EO (39.25%). Several studies have addressed the volatile composition of *C. ladanifer*, with α-pinene being the compound that always presented the highest concentration (i.e., from 30 to 50%) [15]. Furthermore, the composition of this EO is complex, containing more than 90 compounds, including some in trace amounts, as other authors have reported [30].

α-Pinene, a bicyclic monoterpene, is a food additive used as flavoring agent or adjuvant [31] and is Generally Recognized as Safe (GRAS) by Food and Drug Administration (FDA) [32]. This compound is known for its bioactivities, namely its antioxidant [33] and antimicrobial [34] properties.

### 3.2. Antioxidant, Antibacterial and Anti-quorum sensing Properties of Rockrose EO

The antioxidant properties of rockrose EO were evaluated by two methods: the DPPH free radical scavenging assay and β-carotene bleaching test (Table 2). Regarding the results obtained by the DPPH method, rockrose EO showed very strong antioxidant activity (IC_50_ = 0.90 ± 0.10; AAI = 5.73 ± 0.89), yet significantly lower (*p*-value < 0.05) than the antioxidant activity of gallic acid, used as a standard (Table 2). However, the capacity of this EO to inhibit the lipid peroxidation, evaluated by β-carotene bleaching test, was significantly higher (*p*-value < 0.05) than that obtained for BHT (Table 2). BHT is a synthetic antioxidant often used by the food industry, but recent reports revealed that this compound may be implicated in health risks, including cancer and carcinogenesis [35]. Considering the obtained results, it is possible to suggest the potential substitution of BHT with rockrose EO in the food industry. The antioxidant activity of essential oils is of great interest, as they can be used in food to preserve it from oxidation processes, thus prolonging shelf-life without losing nutritional quality attributes [15]. 

The antioxidant potential of rockrose EO may be a result of its phenol rich chemical profile, since phenolic compounds and terpenoids are natural chain breaking antioxidants, as previously reported by other authors [18]. 

Concerning the antibacterial activity of rockrose EO, initially, a screening using the solid diffusion assay was performed (Table 3). Analyzing the diameters of the inhibition zones, it was verified that rockrose EO is more active against the tested Gram-positive bacteria. Furthermore, the result obtained for *L. monocytogenes* LMG 16779 was not significantly different (*p*-value > 0.05) when compared with that of tetracycline (Table 3). Previous studies have indicated that α-pinene, the major constituent of rockrose EO, can be considered as possessing weak activity against the most tested microorganisms [36], suggesting that synergic action may occur between all the compounds of rockrose EO.

The antimicrobial activity of the EO was further evaluated by the determination of its MIC values (Table 4). Confirming the results obtained by solid diffusion assay, the lowest MIC values of rockrose EO were obtained for Gram-positive bacteria, particularly for *L. monocytogenes* LMG 16779, *E. faecalis* ATCC 29212 and *B. cereus* ATCC 11778.

Overall, rockrose EO presented stronger antibacterial activity against Gram-positive than against Gram-negative bacteria. The structures of Gram-negative bacteria are more complex than those of Gram-positive, protecting themselves from the external environment and resisting EO permeation [37].

The ability of bacteria to sense and respond to their population density is named *quorum sensing* [38]. A complex of signaling molecules and receptor proteins triggers the expression of specific genes which are responsible for various phenotypes, including violacein pigment in *Chromobacterium violaceum* [38]. The anti-*quorum sensing* potential of rockrose EO was also studied by solid diffusion assay (Table 3). The capacity of the EO to inhibit violacein production was significantly higher (*p*-value < 0.05) than that observed for resveratrol, which suggests that this EO can interfere with cell-to-cell communication, and therefore, may inhibit *quorum sensing*-mediated biofilm formation, a major cause of bacterial pathogenesis.

### 3.3. FTIR and DSC Analysis of the Films

**Figure 1:**
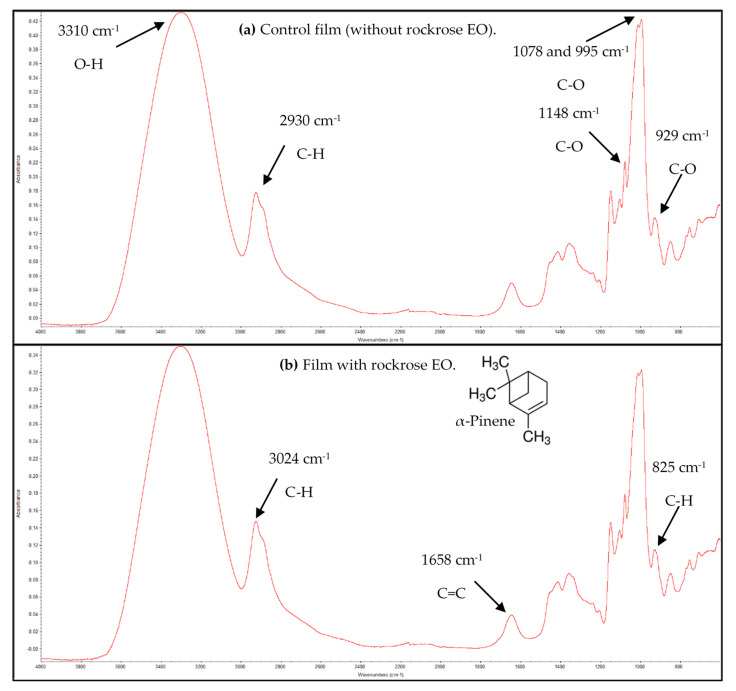
FTIR spectra of the pullulan-based films.

The FTIR spectra of the pullulan-based films were obtained (Figure 1). In the FTIR spectrum of the control film (without rockrose EO) (Figure 1a), the infrared bands corresponding to the stretching vibrations of the O-H groups of the polymer at 3310 cm^−1^ are shown. C-H vibrations appeared at 2930 cm^−1^ and the C-O stretching vibrations of the glycosidic and etheric bounds of the polymer were observed at 1148 cm^−1^, 1078 cm^−1^, 995 cm^−1^ and 929 cm^−1^ [39]. 

The FTIR spectrum of pullulan film containing rockrose EO is shown in Figure 1b. 

Since the major compound of the EO is α-pinene, its characteristic infrared peaks were highlighted, namely, the absorption peak at 3024 cm^−1^ (stretching vibrations due to unsaturated C-H), 885 cm^−1^ (bending vibration due to unsaturated C-H) and 1658 cm^−1^ (stretching vibration of C = C) [40]. As the concentration of rockrose EO is much lower than that of pullulan, the peaks corresponding to α-pinene are superimposed with those of pullulan, as can be seen in Figure 1.

**Figure 2:**
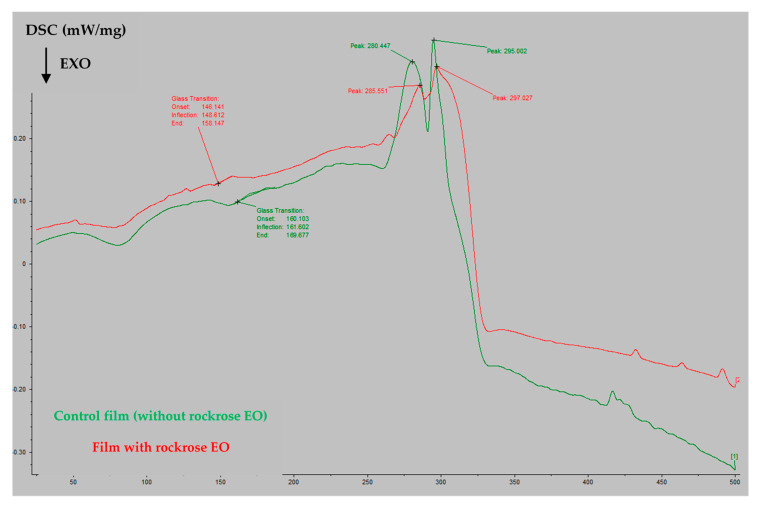
DSC thermograms of the pullulan-based films.

The thermal profiles of pullulan films with or without rockrose EO incorporated were evaluated by DSC (Figure 2). The glass transition temperature (T_g_) of pullulan is 154.5 °C. The T_g_ is the temperature at which a material undergoes a structural transition from an amorphous solid state (glassy state) to a more viscous, rubbery state. Below T_g_, films are rigid and brittle, whereas above T_g_, they become flexible and pliable [41]. The incorporation of rockrose EO into the films slightly decreased their T_g_ from 161.602 °C to 148.612 °C (Figure 2), indicating that the EO will affect the structure of the films, and consequently, their mechanical properties. 

### 3.4. Grammage, Thickness, Mechanical and Optical Properties 

The grammage and thickness of the films significantly increased (*p*-value < 0.05) with the incorporation of rockrose EO (Table 5), similarly to what was previously observed for other pullulan-based films [23], which was also expected, because both the grammage and thickness of the films depend on the total solid content [42].

The mechanical properties of the films are primarily characterized by elongation at break, tensile index and elastic modulus, which depend on the type of polymer matrix, the type of additives and the interactions between them. The typical stress *versus* deformation curves exhibit the mechanical properties of the films more clearly [43]. Concerning the mechanical properties of the pullulan-based films developed in this work (Table 5), they were significantly affected (*p*-value < 0.05) by the incorporation of rockrose EO. The elongation at break, tensile index and elastic modulus decreased in the films containing the EO (Table 5), as expected by the DSC analysis mentioned above. These results may be explained by the heterogeneous film structure featuring discontinuities induced by EO incorporation, as described in a previous study [43]. Similar results were obtained for pullulan films containing cinnamon EO and Tween 80, where the authors explained that the incorporation of EO not only attenuated intermolecular hydrogen bonding between pullulan-pullulan molecules, but also loosened the film structure by allowing EO droplet occupancy, leading to a decrease in the tensile index of the composite films [22].

The optical properties of the films were significantly affected (*p*-value < 0.05) by the incorporation of rockrose EO, except the yellowness (*p*-value > 0.05) (Table 5). The transparency of the films decreased with the addition of the EO, which could be related to the light-scattering caused by the distribution of the EO droplets within the biopolymer matrix [44]. Nonetheless, the transparency of the films containing rockrose EO was 94.66%, a substantial higher value than what was observed for starch-carboxy methyl cellulose films containing rosemary EO, i.e., about 1% [44].

### 3.5. Contact Angles and Surface Free Energies

Water contact angle is the most commonly used method to estimate the wettability or the surface hydrophobicity. A water contact angle higher than 90° indicates that the surface has a considerable hydrophobic nature; when that angle is lower than 90°, more significant hydrophilic properties will occur [45]. The water contact angles of pullulan films are summarized in Table 6. It was observed that the incorporation of rockrose EO significantly increased (*p*-value < 0.05) the water contact angle of the films (from ≈65° to ≈74°), increasing their hydrophobicity, which is of major importance when packaging foods with high moisture contents.

This observation may be explained by the hydrophobic behavior of the EO. Its major compound, α-pinene, presents an octanol/water partition coefficient (logP) of 4.83, suggesting lower polarity, and consequently, its hydrophobic nature.

By measuring the contact angle with three pure liquids (water, ethylene glycol and diiodomethane) presenting different polarities, and applying the Owens-Wendt approach, it was possible to determine the surface free energy of the films (Table 6). The total surface free energy and the respective polar component of the pullulan-based films were significantly lower (*p*-value < 0.05) when the rockrose EO was incorporated, while the dispersive component was significantly higher (*p*-value < 0.05). These results are also associated with the hydrophobicity of rockrose EO, as mentioned earlier. 

Furthermore, the contact angles were measured on both sides of the films (Table 6). The overall results showed no substantial differences between both sides of the same type of film.

### 3.6. Barrier Properties

Food packaging films should create a protective atmosphere around the food product during transportation, handling and commercialization. They should also improve the shelf-life of perishable foods by acting as a good barrier to moisture and gases (CO_2_, O_2_) [46].

Concerning the water vapor permeability of the films (Table 7), it was verified that the incorporation of rockrose EO did not significantly change (*p*-value > 0.05) the values of WVTR and WVP. The entrapment of water inside the polysaccharide-based films has been previously explained, and is related to possible interactions among polysaccharide molecules and the hydroxyl groups of glycerol, leading to a more compact polymeric network [47].

Contrariwise to what was observed for water vapor permeability, the incorporation of rockrose EO in the pullulan films significantly increased (*p*-value < 0.05) their oxygen permeability (Table 7), which is probably linked to the plasticizing effect of the EO, as other authors reported when adding carvacrol to biopolymeric composite films [48]. Further studies will be needed to better understand the increase in oxygen permeability of the pullulan films when rockrose EO is incorporated, and to overcome this drawback.

### 3.7. Antioxidant, Antibacterial and Anti-quorum sensing Properties of Bioactive Films

**Figure 3:**
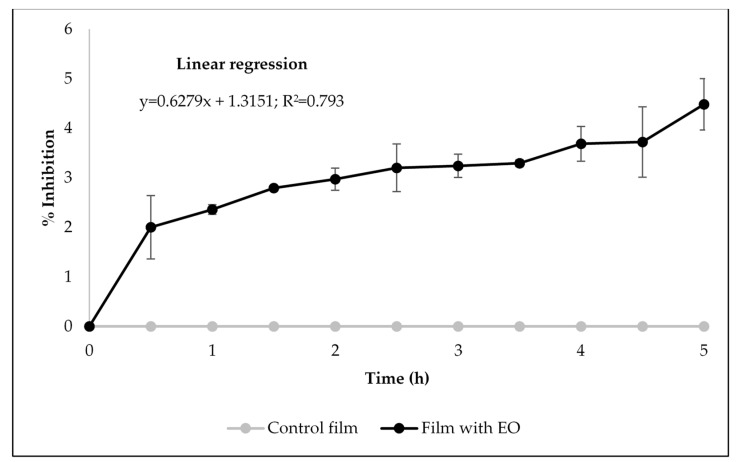
Antioxidant activity of the films measured by DPPH free radical scavenging assay.

Food products are often deteriorated by free radical-mediated oxidation of unsaturated lipids during storage, leading to rancidity, nutritional decrement, and the generation of toxic compounds [18]. For that reason, the antioxidant activity of the developed films was evaluated. Figure 3 shows the results obtained with the DPPH free radical scavenging assay, which allowed to conclude that the control film (without rockrose EO) presented no capacity to scavenge DPPH radicals; in contrast, the pullulan film containing the EO demonstrated such a capacity, which was shown to be time-dependent, as the linear regression suggests (R^2^ = 0.793) (Figure 3). The antioxidant activity of rockrose EO is maintained when it is incorporated within the film matrix, indicating its release into the reaction mixture. Moreover, the degree of antioxidant power of edible films is generally proportional to the amount of the antioxidant additives, as previously described [49].

Bearing in mind the potential application of the films developed in this study to package foods with high amounts of fat, their antioxidant activity was further evaluated by a β-carotene bleaching test (Table 8).

Interestingly, the results showed no significant differences (*p*-value < 0.05) between the capacity of the pullulan-based films to inhibit lipid peroxidation when the rockrose EO was incorporated (≈73%) (Table 8). Similar results were achieved previously in a study dealing with gelatin-chitosan-pectin films incorporated with rosemary EO, where the percentage of inhibition determined by β-carotene bleaching test for both types of films (control and with EO) was near to 22% [50], a value considerably lower than that now obtained. These overall results indicate the potential of the developed films to be used to package foods which are susceptible to oxidation and rancification to improve their shelf-life.

Since rockrose EO presented antibacterial activity, particularly against some Gram-positive, food-borne bacteria, the antibacterial properties of the pullulan-based films were also evaluated (Table 8). It was verified that the incorporation of the EO in the pullulan films caused bacterial cell retraction at the contact area with a clear inhibition zone for Gram-positive species, in contrast to what was observed for Gram-negative bacteria and for the control film (without rockrose EO), where bacterial growth occurred on the top of the films (Table 8). Gram-negative bacteria are generally more resistant than Gram-positive; this may be attributed to their outer membrane being impermeable to lipophilic compounds [51].

Regarding the anti-*quorum sensing* activity of the films, it was only observed when rockrose EO was incorporated into the films (Table 8). The results obtained in this study reflect the potential of rockrose EO, free or incorporated in pullulan, as a promising *quorum sensing* inhibitor, since it was able to interrupt intercellular communication, thereby inhibiting violacein production. Similar results were obtained for oregano EO alone or when incorporated into pectin films [52].

### 3.8. Antibiofilm Activity

**Figure 4:**
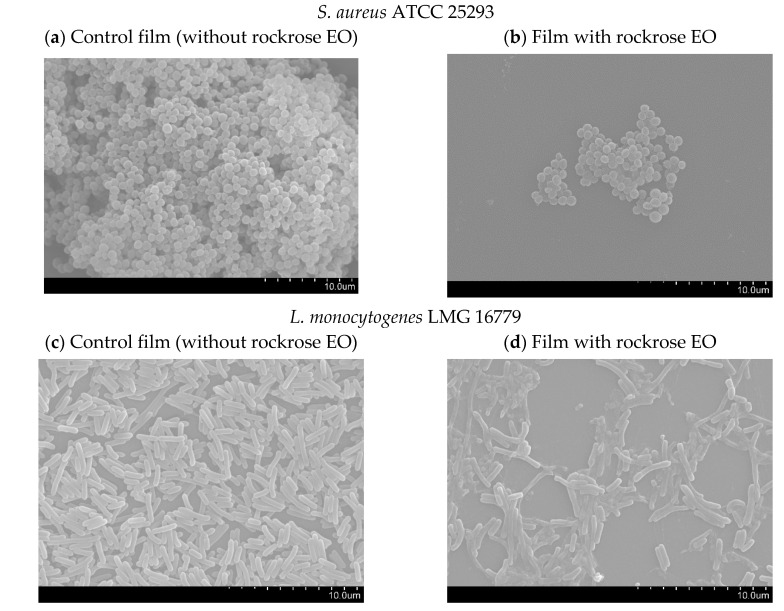

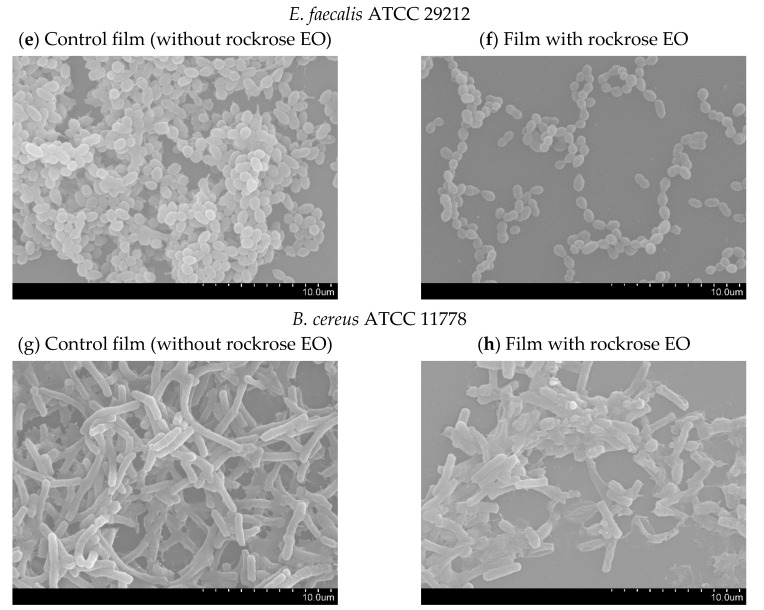
SEM micrographs (**a**)–(**h**) of the Gram-positive bacteria biofilms formed directly on the surface of the pullulan-based films (Magnification: 5000×).

Considering that rockrose EO presented the best antibacterial activity against the Gram-positive bacteria tested, as also verified when it was incorporated in pullulan films, together with the anti-*quorum sensing* action of the free and incorporated EO, the antibiofilm activity of the developed films was additionally evaluated. For that, the tested Gram-positive bacteria biofilms were formed directly on the surface of the films, which were then observed by SEM (Figure 4).

The *S. aureus* ATCC 25923 biofilm formed on the control film (without rockrose EO) (Figure 4a) was constituted by several layers of cells, but when the EO was incorporated, there was no biofilm formation (Figure 4b). Similar results were achieved for *L. monocytogenes* LMG 16779, that formed a typical three-dimensional biofilm on the surface of the control film (Figure 4c), while a minimal biofilm was formed when rockrose EO was incorporated (Figure 4d). The biofilm of *E. faecalis* ATCC 29212 on the control film was abundant, allowing the bacteria to connect to each other (Figure 4e). This bacterial growth and biofilm formation were reduced when *E. faecalis* ATCC 29212 were placed on a pullulan film containing rockrose EO (Figure 4f). The cells of *B. cereus* ATCC 11778 appeared to be destroyed when grown on the film with EO (Figure 4g), in contrast to what was verified using the control film (Figure 4h).

These results clearly indicate the antibiofilm activity of the films with rockrose EO, which was able to influence bacterial adhesion. This may be explained by the differences in the surface free energy of the films, as mentioned earlier.

## 4. Conclusions

This work demonstrated the antioxidant, antibacterial and anti-*quorum sensing* properties of rockrose EO, and showed that these bioactivities are maintained after its incorporation in pullulan-based films. The developed films were fully characterized and may be used as a new active packaging material, contributing to improving the shelf-life of packaged foods and preventing biofilm formation.

## Figures and Tables

**Table 1:** Chemical composition of rockrose EO.

Retention Time (min)	Compound	% Relative
6.42	1,2,3-Trimethylcyclopentene	0.13
6.65	1,3-Dimethylcyclohexene	0.02
9.73	1,2,4,4-Tetramethylcyclopentene	0.63
10.64	*trans*-Pinane	0.12
13.03	Tricyclene	1.56
13.93	α-Pinene	39.25
14.05	α-Thujene	0.24
14.71	Toluene	0.03
15.93	α-Fenchene	0.15
16.48	Camphene	8.22
19.14	β-Pinene	0.57
20.02	Sabinene	0.29
20.25	Verbenene	0.87
22.86	β-Myrcene	0.11
23.14	α-Phellandrene	0.12
24.17	α-Terpinene	0.37
25.29	Menthatriene isomer	0.14
25.59	Limonene	1.61
26.34	1,8-Cineole	0.29
26.46	β-Phellandrene	0.88
26.75	1,2,3-*p*-Menthatriene	0.15
28.86	Dehydroxy-*cis*-linalool oxide	0.09
29.01	γ-Terpinene	0.92
29.28	*p*-Mentha-1,5,8-triene	0.08
29.57	Styrene	0.02
30.81	*p*-Cymene	3.47
31.76	α-Terpinolene	0.25
32.45	*p-*Cymenyl	0.07
33.51	Unknown alcohol	0.32
34.61	2,2,6-Trimethylcyclohexanone	3.20
34.96	Pinol	0.10
37.00	*cis*-Rose oxide	0.08
38.12	*trans*-Rose oxide	0.03
40.58	Bornyl chloride	0.34
40.75	Isophorone isomer	0.28
42.20	α-Campholenal	0.21
42.87	α-*p*-Dimethylstyrene	0.51
44.66	α-Cubenene	0.07
44.96	Vitispirane	0.43
46.75	α-Ylangene	0.48
46.85	Unknown sesquiterpene	0.70
47.39	α-Copaene	0.57
47.82	Unknown alcohol	0.15
48.22	3-Nonen-2-one	0.22
48.50	Unknown alcohol	0.30
48.95	Camphor	0.83
49.67	Unknown sesquiterpene	0.03
49.88	α-Gurjunene	0.05
49.93	Linalool	0.35
51.00	Isopinocamphone	1.04
52.36	Pinocarvone	0.77
53.07	Bornyl acetate	5.06
54.15	Terpinen-4-ol	1.42
54.52	*trans*-β-Caryophyllene	0.32
54.99	Aromadendrene	0.08
55.28	Unknown sesquiterpene	0.02
55.75	β-Cyclocitral	0.06
56.33	Myrtenal	0.83
57.20	Unknown sesquiterpene	0.18
57.48	*trans*-Pinocarveol	5.48
57.75	Allo-aromadendrene	1.10
57.97	α-Phellandren-8-ol (I)	0.05
58.25	*trans*-Cadina-1(6),4-diene	0.12
59.81	Myrtenyl acetate	0.46
60.00	α-Terpineol	0.19
60.23	α-Amorphene	0.11
60.33	Borneol	1.25
60.71	Dehydro-aromadendrane	0.08
60.78	Ledene	0.33
61.46	Verbenone	0.26
61.63	β-Himachalene + α-Phellandren-8-ol (II)	0.25
62.53	Carvyl acetate	0.16
62.67	Carvone	0.14
64.31	Δ-Cadinene	0.58
65.73	Myrtenol	0.71
65.89	Unknown alcohol	0.11
68.10	*trans*-Carveol	0.31
68.95	*trans*-Calamenene	0.23
73.74	α-Calacorene	0.23
74.80	Palustrol	0.11
77.48	Unknown sesquiterpenol	0.11
79.95	Ledol	0.64
80.87	Cubeban-11-ol	0.03
82.19	Viridiflorol	2.04
83.68	Spathulenol	0.11
84.73	Eugenol	0.05
86.05	Unknown sesquiterpenol	0.21
86.30	Junenol	0.05
86.66	Ambrox	0.08
88.45	β-Eudesmol	0.04
98.47	Unknown diterpene	0.06
**Total identified**		**95.36**
Monoterpenes		51.00
Monoterpenoids		22.53
Cyclic unsaturated		4.10
Sesquiterpenoids		3.48
Sesquiterpenes		3.15
Others		11.10

**Table 2:** Antioxidant properties of rockrose EO.

Method	Parameters	Essential Oil	Gallic Acid	*p*-Value	BHT	*p*-Value
DPPH	IC_50_ (%)	0.90 ± 0.10	0.23 ± 0.01	0.007 *	-	-
	AAI	5.73 ± 0.89	22.56 ± 0.20	0.001 *	-	-
	Antioxidant activity	Very strong	Very strong	-	-	-
β-carotene bleaching	IC_50_ (%)	0.48 ± 0.04	-	-	3.58 ± 0.02	<0.001 *

Results expressed as mean ± SD; * indicates a significant result (*p*-value < 0.05).

**Table 3:** Diameters of inhibition zones and of inhibition of the violacein production (mm).

Microorganisms	Essential Oil (15 µL/disc) ^a^	Tetracycline (30 µg/disc) or Resveratrol (5 µg/disc) ^b^	DMSO (15 µL/disc) ^c^	*p*-Values
*S. aureus* ATCC 25923	18.84 ± 0.58	30.65 ± 1.92	6.00 ± 0.00	0.005 ^ab,^* 0.001 ^ac,^*
*L. monocytogenes* LMG 16779	17.01 ± 0.06	18.45 ± 0.69	6.00 ± 0.00	0.068 ^ab^ <0.001 ^ac,^*
*E. faecalis* ATCC 29212	13.77 ± 1.17	25.30 ± 2.00	6.00 ± 0.00	0.002 ^ab,^* 0.007 ^ac,^*
*B. cereus* ATCC 11778	18.28 ± 0.45	30.60 ± 1.95	6.00 ± 0.00	0.006 ^ab,^* <0.001 ^ac,^*
*E. coli* ATCC 25922	11.01 ± 1.34	23.84 ± 0.72	6.00 ± 0.00	0.001 ^ab,^* 0.023 ^ac,^*
*S.* Typhimurium ATCC 13311	7.80 ± 1.00	19.63 ± 1.01	6.00 ± 0.00	<0.001 ^ab,^* 0.089 ^ac^
*P. aeruginosa* ATCC 27853	6.54 ± 0.76	11.56 ± 0.86	6.00 ± 0.00	0.002 ^ab,^* 0.344 ^ac^
*C. violaceum* ATCC 12472	9.69 ± 0.44	8.49 ± 0.20	0.00 ± 0.000	0.027 ^ab,^* 0.001 ^ac,^*

Results expressed as mean ± SD; upper letters (a, b, c) were used to identify the pairs of samples under statistical comparison; * indicates a significant result (*p*-value < 0.05).

**Table 4:** MIC values of rockrose EO.

Microorganisms	Essential Oil (µL/mL)	Tetracycline (µg/mL)	DMSO (%, *v/v*)
*S. aureus* ATCC 25923	16	0.06	>2
*L. monocytogenes* LMG 16779	8	0.06	>2
*E. faecalis* ATCC 29212	8	0.06	>2
*B. cereus* ATCC 11778	2	0.06	>2
*E. coli* ATCC 25922	32	0.06	>2
*S.* Typhimurium ATCC 13311	32	0.24	>2
*P. aeruginosa* ATCC 27853	32	0.24	>2

Results expressed as modal values.

**Table 5:** Grammage, thickness, mechanical and optical properties of the films.

Properties		Control Film (without Rockrose EO)	Film with Rockrose EO	*p*-Value
Grammage (g/m^2^)		83.44 ± 0.64	87.17 ± 0.90	0.006 *
Thickness (µm)		54.11 ± 2.72	61.80 ± 0.63	0.034 *
Mechanical properties	Elongation at break (%)	3.15 ± 0.17	2.34 ± 0.07	0.007 *
	Tensile index (N.m/g)	37.33 ± 1.78	24.73 ± 1.22	0.001 *
	Elastic modulus (MPa)	2881.40 ± 102.27	2244.42 ± 173.71	0.010 *
Optical properties	L* (lightness)	21.94 ± 0.34	28.00 ± 0.53	<0.001 *
	a* (redness)	−0.17 ± 0.02	−0.24 ± 0.01	0.013 *
	b* (yellowness)	−1.26 ± 0.11	−1.18 ± 0.06	0.347
	Transparency (%)	96.28 ± 0.22	94.66 ± 0.14	0.001 *

Results expressed as mean ± SD; * indicates a significant result (*p*-value < 0.05).

**Table 6:** Contact angles and surface free energy (*ɤ*) of the films.

Properties	Control Film (without Rockrose EO)		Film with Rockrose EO		*p*-Values
	Lower Side ^a^	Upper Side ^b^	Lower Side ^c^	Upper Side ^d^	
Water contact angle (°)	66.17 ± 1.79	64.99 ± 3.11	77.01 ± 2.10	70.58 ± 2.11	0.003 ^ac,^* 0.070 ^bd^
Ethylene glycol contact angle (°)	59.07 ± 1.96	49.94 ± 0.79	71.72 ± 2.70	63.77 ± 1.32	0.004 ^ac,^* <0.001 ^bd,^*
Diiodomethane contact angle (°)	31.43 ± 1.33	37.06 ± 0.44	32.81 ± 0.79	36.74 ± 1.20	0.213 ^ac^ 0.699 ^bd^
Total surface free energy, ɤ^T^ (mN/m)	40.93 ± 2.05	43.11 ± 2.16	33.48 ± 1.67	37.49 ± 1.87	0.009 ^ac,^* 0.028 ^bd,^*
Dispersive component, ɤ^D^ (mN/m)	13.29 ± 0.66	11.85 ± 0.59	22.95 ± 1.15	25.79 ± 1.29	0.001 ^ac,^* 0.001 ^bd,^*
Polar component, ɤ^P^ (mN/m)	27.63 ± 1.38	31.26 ± 1.56	10.53 ± 0.53	11.70 ± 0.59	0.001 ^ac,^* 0.001 ^bd,^*

Results expressed as mean ± SD; upper letters (a, b, c, d) were used to identify the pairs of samples under statistical comparison; * indicates a significant result (*p*-value < 0.05).

**Table 7:** Barrier properties of the films.

Permeability		Control Film (without Rockrose EO)	Film with Rockrose EO	*p*-Value
Water vapor	WVTR (g/m^2^.day)	29.14 ± 3.38	30.77 ± 3.66	0.601
	WVP (g/Pa.day.m) (×10^−6^)	1.19 ± 0.14	1.44 ± 0.17	0.123
Oxygen	OTR (cm^3^/m^2^.day)	5038.32 ± 215.92	12820.78 ± 1160.76	0.006 *
	OP (cm^3^.µm/m^2^.day.kPa)	2971.00 ± 56.57	8160.00 ± 226.98	<0.001 *

Results expressed as mean ± SD; * indicates a significant result (*p*-value < 0.05).

**Table 8:** Antioxidant activity (β-carotene bleaching test), antibacterial properties and diameter of inhibition of the violacein production (mm).

Properties		Control Film (without Rockrose EO)	Film with Rockrose EO	*p*-Value
**β-carotene bleaching test**	% Inhibition	69.67 ± 1.75	76.52 ± 3.90	0.076
Antibacterial	*S. aureus* ATCC 25923	0.00 ± 0.00 (-)	6.00 ± 0.00 (+)	<0.001 *
	*L. monocytogenes* LMG 16779	0.00 ± 0.00 (-)	6.00 ± 0.00 (+)	<0.001 *
	*E. faecalis* ATCC 29212	0.00 ± 0.00 (-)	6.00 ± 0.00 (+)	<0.001 *
	*B. cereus* ATCC 11778	0.00 ± 0.00 (-)	6.00 ± 0.00 (+)	<0.001 *
	*E. coli* ATCC 25922	0.00 ± 0.00 (-)	0.00 ± 0.00 (-)	<0.001 *
	*S.* Typhimurium ATCC 13311	0.00 ± 0.00 (-)	0.00 ± 0.00 (-)	<0.001 *
	*P. aeruginosa* ATCC 27853	0.00 ± 0.00 (-)	0.00 ± 0.00 (-)	<0.001 *
Inhibition zone (mm)	*C. violaceum* ATCC 12472	0.00 ± 0.00	6.00 ± 0.00	<0.001 *

Results expressed as mean ± SD; (-) bacterial growth on the top of the films; (+) bacterial cells retraction at the contact area with a clear inhibition zone; * indicates a significant result (*p*-value < 0.05).
